# Integration of tongue image features and tongue coating microbiome for differentiating dampness patterns in MASLD

**DOI:** 10.3389/fendo.2026.1851610

**Published:** 2026-05-22

**Authors:** Ruomeng Hu, Jialin Deng, Liping Tu, Tao Jiang, Jiatuo Xu

**Affiliations:** School of Traditional Chinese Medicine, Shanghai University of Traditional Chinese Medicine, Shanghai, China

**Keywords:** Machine learning, metabolic dysfunction-associated steatotic liver disease (MASLD), TCM pattern, tongue coating microbiome, tongue diagnosis

## Abstract

**Background:**

Metabolic dysfunction-associated steatotic liver disease (MASLD) is a prevalent metabolic disorder that can progress to hepatic fibrosis, cirrhosis, and hepatocellular carcinoma. Traditional Chinese medicine (TCM) has therapeutic effects in MASLD. In TCM, dampness patterns predominate in MASLD and are further classified into damp heat (DH) and cold dampness (CD) subtypes based on distinct pathogenic mechanisms. However, the characteristics of the tongue image and tongue coating microbiome across different dampness patterns and their associations with clinical features remain poorly understood.

**Materials and methods:**

This study enrolled 320 participants, including 214s dampness patterns patients with MASLD, further classified into DH pattern (n = 110) and CD pattern(n = 104), along with 106 healthy controls. Tongue images and tongue coating samples were collected; tongue image segmentation was performed using a customized Tongue-InSPyReNet framework and extract quantitative features, while tongue coating microbiota were profiled using 16S rRNA gene sequencing. To distinguish between patterns, five machine learning models were developed and evaluated based on integrated tongue image features and microbiome data.

**Results:**

Significant differences in tongue features and microbial composition were observed between groups. The DH pattern was characterized by a red-crimson tongue with yellow coating and enrichment of *Prevotella* (P < 0.05). In contrast, the CD pattern more frequently exhibited a pale tongue with petechiae and higher abundances of *Streptococcus* and *Rothia* (P < 0.05). Integration of tongue image features and tongue coating microbiome effectively distinguished DH and CD patterns, achieving an AUC of 0.871 and an accuracy of 79.1%.

**Conclusion:**

Our study highlights the contributions of tongue image features and the tongue coating microbiome to differentiating two TCM patterns in MASLD, and may provide the rationale for adopting different treatment strategies for different TCM syndromes of MASLD in the future.

## Introduction

Metabolic dysfunction-associated steatotic liver disease (MASLD) is a key component of metabolic diseases, characterized by a spectrum ranging from simple steatosis to non-alcoholic steatohepatitis (NASH) and fibrosis (1, 2). As a prevalent chronic liver disease, MASLD is estimated to affect more than one-third of the global adult population (3). It can progress to fibrosis, cirrhosis, and hepatocellular carcinoma, and is closely associated with extrahepatic complications, including cardiovascular disease and chronic kidney disease (4). Traditional Chinese medicine (TCM) has advantages in alleviating the clinical symptoms of patients with liver diseases and improving liver function (5). TCM treatment is guided by pattern differentiation, pattern represents a comprehensive summary of pathological characteristics at a specific stage of disease (6). However, the symptoms used for pattern differentiation are difficult to quantify, limiting the objectivity and reproducibility of TCM diagnosis (7). Therefore, it is essential to explore the quantitative and valid biological scientific foundation of patterns to accurate TCM diagnosis and modernization.

In traditional TCM theory, MASLD is generally categorized as a form of “liver disease”. Due to the existence of multiple etiological factors (8), MASLD can present different TCM pattern types (9). Previous studies have shown that dampness-related patterns are the predominant TCM syndromes in MASLD (10, 11), accounting for approximately 30%–40% of patients (11), and are closely associated with disturbances in water–fluid metabolism and metabolic dysfunction (12). According to patterns differentiation based on heat and cold in TCM, it can be classified into Damp heat (DH) pattern and Cold dampness (CD) pattern (6). They reflect different pathological mechanisms and should be differentiated and treated with different methods (13). However, at present, there is a lack of understanding of the material basis of these two types of dampness syndromes, and the diagnostic methods are difficult to quantify.

Tongue diagnosis is the cornerstone of TCM diagnostics (14), the tongue body reflects the functional state of internal organs, while the tongue coating reveals the depth and essence of pathological influences (15, 16). TCM proposes a close association among tongue image, pattern, and disease progression. However, traditional tongue diagnosis has shortcomings such as strong subjectivity. Therefore, it becomes very necessary to use objective tongue image features as a method for pattern classification. Recent advances have enabled the integration of modern tongue diagnosis with computer science, deep learning models enabling large-scale tongue image analysis and comprehensive pathological interpretation (17). The tongue coating harbors a complex microbial community that may contribute to tongue feature formation (18). Our previous study showed that integrating tongue image features with tongue coating microbiota improves pattern classification (9). However, the relationship between tongue image characteristics and the tongue coating microbiome in Dampness patterns remains unclear.

According to TCM theory, the same disease may present with different patterns, necessitating pattern differentiation individualized therapeutic strategies. Dampness is the most prevalent etiological factor of MASLD. However, there is still a lack of objective evidence to distinguish different dampness patterns in MASLD. This study focuses on the two most prevalent dampness patterns in MASLD, Damp heat pattern and Cold dampness pattern, and explores variations in tongue image features and tongue coating microbiome across TCM patterns, aiming to provide a basis for accurate diagnosis of MASLD, facilitate personalized treatment, and further enhance the understanding of the scientific basis underlying TCM pattern differentiation.

## Data and methods

### Study design and subjects

**Figure 1:**
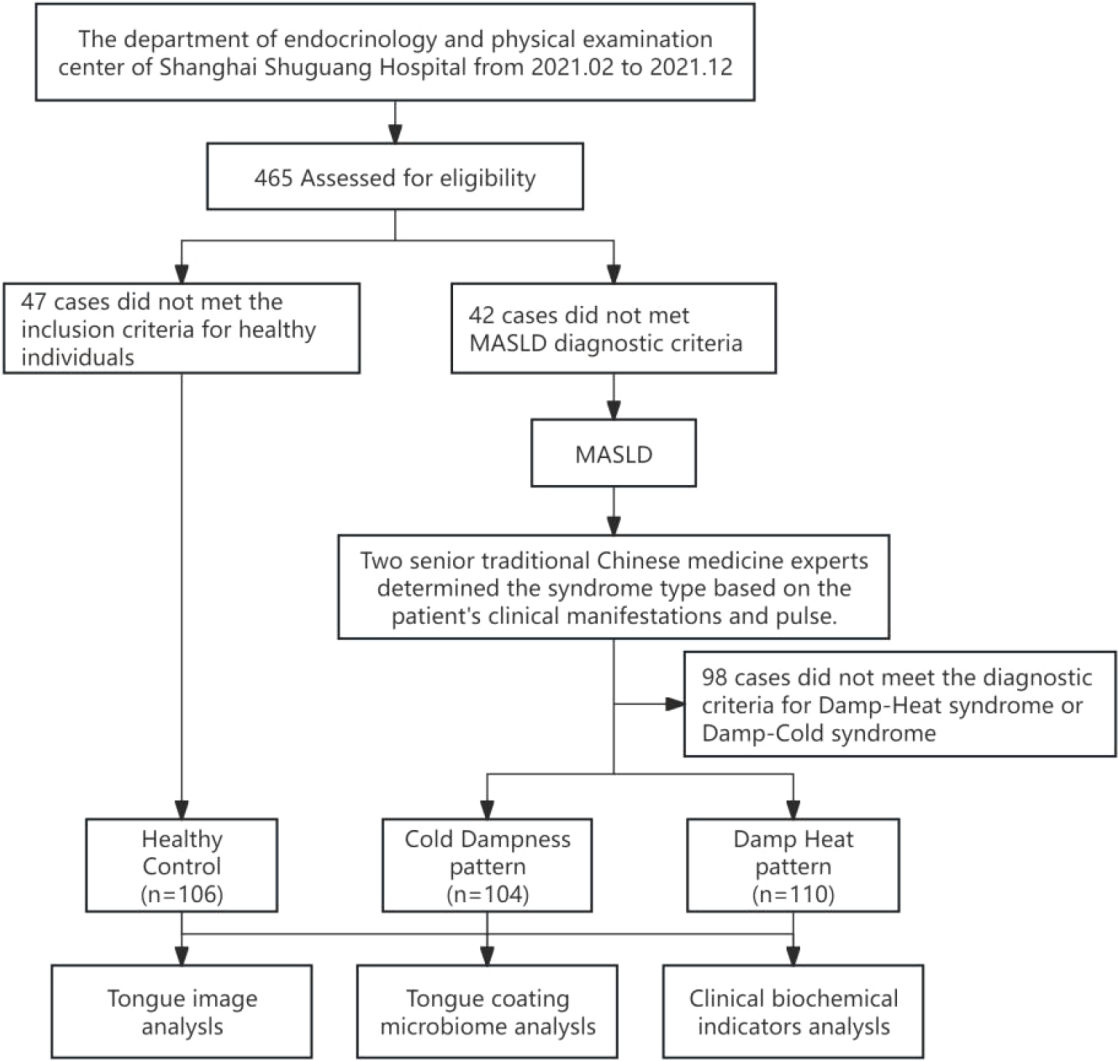
Program flowchart.

The overall study design is illustrated in **Figure 1**. The study population comprised patients who attended the Department of Endocrinology and the Physical Examination Center at Shuguang Hospital, affiliated with Shanghai University of Traditional Chinese Medicine, between February and December 2021. According to the *Expert Consensus on the Diagnosis and Treatment of Non-alcoholic Fatty Liver Disease with Integrated Traditional Chinese and Western Medicine (2025) (*19), senior experts classified the patients into pattern types based on clinical symptoms, tongue appearance and pulse. Finally, patients with Damp-Heat pattern and cold dampness pattern of MASLD were selected as the subsequent research subjects. The inclusion and exclusion criteria are shown in **Table 1**. A total of 106 healthy volunteers and 214 MASLD patients (110 cases of Damp-Heat pattern and 104 cases of cold dampness pattern) were finally included. All subjects signed the informed consent form approved by the Ethics Committee of Shuguang Hospital Affiliated to Shanghai University of Traditional Chinese Medicine after fully understanding the research purpose, operation process, potential risks and benefits.

**Table 1:** Inclusion and exclusion criteria.

Inclusion criteria	
Age 25–80 years. MASLD Diagnostic Criteria (20): Based on evidence of hepatic fat deposition (histology, noninvasive biomarkers, or imaging), in the presence of at least one of the following conditions: (1) overweight or obesity; (2) type 2 diabetes mellitus; or (3) at least two metabolic dysfunction features.	
Exclusion criteria	
Fatty liver disease or liver damage attributable to medications, excessive alcohol consumption, viral infections, autoimmune disorders, or other identifiable causes; Concurrent presence of liver cancer or other malignancies; Co-existing severe cardiovascular, cerebrovascular, pulmonary, renal, or active malignant conditions, or other organ dysfunction that would impede participation; Severe intestinal diseases such as irritable bowel pattern, ulcerative colitis, or lower gastrointestinal bleeding; Oral diseases, including untreated oral abscesses or fungal infections; Tongue coating condition affected by recent food intake or medication use, potentially confounding TCM diagnostic assessment; A history of probiotic or antibiotic use within the past month; Did not meet the diagnostic criteria for Damp-Heat pattern or cold dampness pattern.	
TCM main pattern evaluation scheme for MASLD (6, 19):	
Damp Heat pattern	Distending pain in the right hypochondriac region; Sticky and unformed stools with difficulty in evacuation; Fullness, distension, or pain in the epigastric and abdominal regions; Red tongue with a yellow, greasy coating, along with a wiry-slippery or soft-rapid pulse.
Cold Dampness pattern	Discomfort or distension in the right hypochondrium; Generalized heaviness and fatigue; Obesity; Sticky and unformed stools with a sensation of incomplete evacuation.

### Data collection and analysis

#### Clinical data collection

Data collected from the subjects comprised the following variables: demographic features including age, gender, body mass index (BMI), Waist-to-Hip Ratio (WHR), Systolic Blood Pressure (SBP), Diastolic Blood Pressure (DBP), 2-hour Plasma Glucose (2hPG), Fasting Plasma Glucose (FPG), Fasting Insulin (FINS), Homeostasis Model Assessment of Insulin Resistance (HOMA-IR), Glycated Hemoglobin (HbA1c), Total Cholesterol (TC), Triglycerides (TG), High-Density Lipoprotein Cholesterol (HDL-C), Low-Density Lipoprotein Cholesterol (LDL-C), Total Bilirubin (TBil), Direct Bilirubin (DBil), Indirect Bilirubin (IBil), Alanine Aminotransferase (ALT), Aspartate Aminotransferase (AST), Alkaline Phosphatase (ALP), Gamma-Glutamyl Transferase (GGT), Total Protein (TP), Creatinine (Cr), Uric Acid (UA), Estimated Glomerular Filtration Rate (eGFR).

#### Tongue image collection

**Figure 2:**
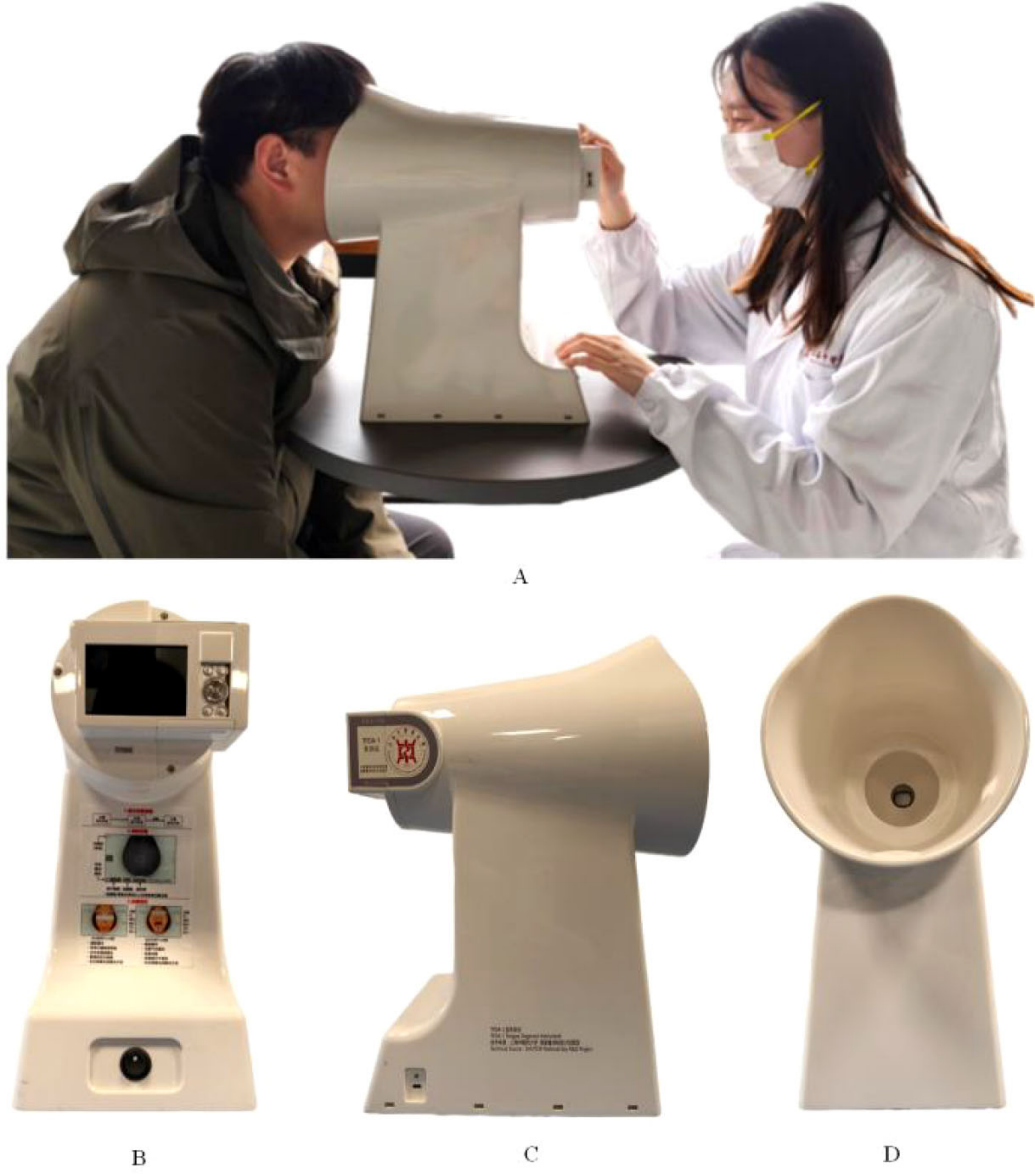
The TFDA-1 tongue diagnostic instrument. **(A)** Operating procedure; **(B–D)** structural diagrams of the TFDA-1 device.

Tongue images were obtained using the TFDA-1 Tongue Diagnostic Instrument developed by the Intelligent Diagnostic Laboratory of Shanghai University of Traditional Chinese Medicine. The specific operating procedure and instrument appearance are shown in **Figure 2**. Image acquisition was performed under controlled room conditions, with the device calibrated using preset parameters and disinfected with alcohol before each use. Participants were instructed to avoid eating, smoking, or consuming colored beverages for at least 15 minutes and to rinse the mouth with warm water. During acquisition, subjects were positioned with the chin supported, the mouth naturally opened, and the tongue relaxed and flattened with the tip slightly downward. All tongue images underwent standardized quality control using a previously developed ResNet-152 based classification model. Only images meeting predefined quality criteria, including complete tongue structure, correct positioning, clear focus, and proper exposure, were included in the study, ensuring overall tongue image data reliability (21).

#### Tongue image diagnosis

**Figure 3:**
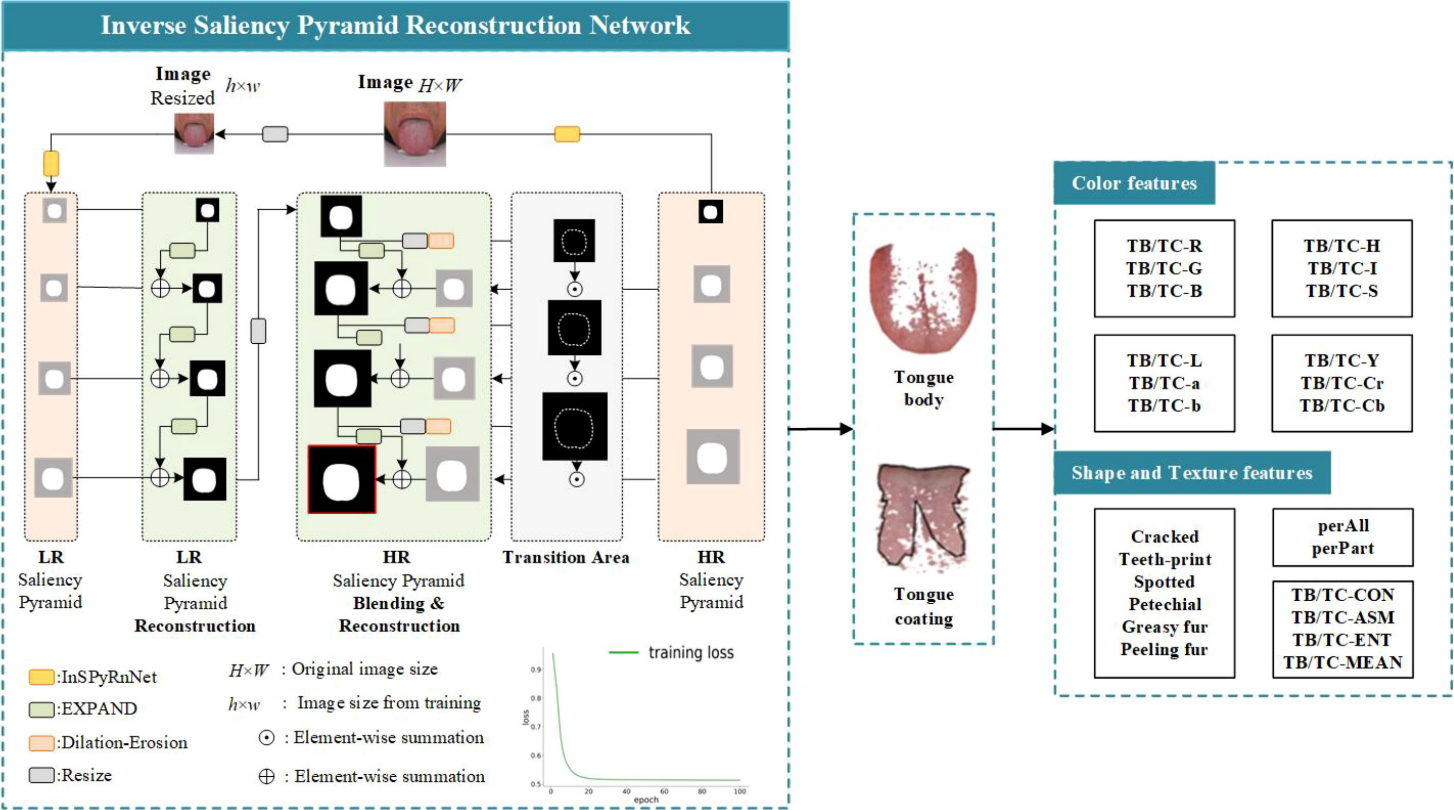
Intelligent analysis of tongue images using the tongue-InSPyReNet model.

InSPyReNet enhances the model’s adaptability to complex tissue structures and improves the delineation of ambiguous boundaries (22). We propose a novel Tongue-InSPyReNet framework that establishes a dedicated analytical pipeline for tongue image segmentation and feature extraction, and the overall workflow is illustrated in **Figure 3**.

A total of 1,200 high-quality tongue images were selected from a standardized database and manually annotated to obtain fine-grained labels of the tongue body and coating, particularly in regions with subtle intensity variations and indistinct boundaries. The dataset was split into 1,000 images for training and 200 for testing.

The proposed Tongue-InSPyReNet was employed for segmentation, leveraging a pyramid reconstruction architecture with Laplacian-based blending to integrate low-resolution semantic information and high-resolution spatial details, thereby alleviating effective receptive field discrepancies. The model was optimized using Adam with a learning rate of 0.00001, trained for 60 epochs with a batch size of 6, and incorporated image resizing and data augmentation strategies to improve robustness and generalization. Experimental results demonstrated that the proposed model achieved an mIoU of 95.20%, mean ACC of 0.989, F-measure of 0.969, MAE of 0.001, and Dice coefficient of 97.5% on the test set, indicating robust performance in fine-grained feature extraction and high-precision segmentation. Based on the segmentation results, quantitative features, including Lab color space values, texture indicators, and tongue coating indices, were further extracted using established pattern recognition methods (23–25).

#### Tongue coating microbiome collection and analysis

Tongue coating samples were collected from all participants between 7:00 and 9:00 a.m. following an overnight fast (≥12 h) to minimize short-term dietary influences on oral microbiota composition. If food had been consumed, participants were instructed to rinse their mouths 2–3 times with normal saline prior to collection and fast for an additional 1–2 h. Samples were obtained by gently rotating a sterile throat swab at least ten times across the mid-posterior region of the tongue dorsum. The swab head was immediately placed into a sterile 2 mL tube, transported on ice, and stored at −80 °C until further processing.

Bacterial DNA was extracted using commercial kits, followed by PCR amplification of the 16S rRNA gene and library preparation according to standard protocols. High-throughput sequencing was performed on the Illumina NovaSeq 6000 platform. Raw sequencing data were processed using the QIIME2 pipeline with the DADA2 algorithm, including demultiplexing, primer trimming, quality filtering, denoising, merging, and chimera removal. Amplicon sequence variants (ASVs) were aligned using MAFFT, and a phylogenetic tree was subsequently constructed using FastTree2. Taxonomic assignment was performed with a naïve Bayes classifier trained on the Greengenes database.

Alpha diversity indices at the ASV level (Chao1, Shannon, Simpson, Faith’s phylogenetic diversity, and Pielou’s evenness) were compared among groups using the Kruskal–Wallis test with Dunn’s *post hoc* test. Beta diversity was assessed based on Aitchison distance derived from centered log-ratio (CLR)-transformed genus-level abundance data and visualized using principal coordinate analysis (PCoA). Group differences were evaluated using permutational multivariate analysis of variance (PERMANOVA), and the homogeneity of group dispersions was assessed using betadisper analysis. Microbial composition at the phylum and genus levels was analyzed. Taxa annotated as unclassified or unidentified at the genus level were excluded prior to downstream analyses. Differentially abundant taxa were identified using ALDEx2, which accounts for the compositional nature of microbiome data through CLR transformation and Dirichlet-multinomial modeling. Furthermore, microbial functional profiles were predicted using PICRUSt2 based on the MetaCyc database.

### Machine learning methods

We employed five complementary machine learning models: Logistic Regression as an interpretable linear baseline; SVM for high-dimensional, small-sample data with non-linear boundaries; and tree-based ensembles (Random Forest, XGBoost, LightGBM) to capture complex interactions, with gradient boosting providing better regularization and predictive performance. For Logistic Regression, L2 regularization was applied using the default sklearn settings, with a maximum number of iterations (max_iter) of 5000 and class_weight set to “balanced”. Tongue image features and microbial data were screened for pattern classification prediction, and all available features were included in model construction. Model performance was robustly evaluated using five-fold stratified cross-validation, in which the dataset was divided into five subsets and performance metrics, including area under the curve (AUC), accuracy, sensitivity, and specificity, were averaged across folds. To capture both linear and non-linear relationships, machine learning analyses were conducted in Python 3.10.9, and classification performance was evaluated using the scikit-learn library (version 1.3.1). Finally, we report the key parameters of the five models and compare their performance.

### Statistical analysis

Statistical analyses were conducted using SCDS v. 25.0 software (IBM Corp., Armonk, NY, USA). The normality of data distribution was assessed using the Shapiro–Wilk test, and homogeneity of variances was evaluated with Levene’s test. Overall comparisons among the control, DH, and CD groups were performed using one-way ANOVA or the Kruskal–Wallis test depending on data distribution, followed by pairwise comparisons when appropriate. Categorical variables were compared using the chi-square test or Fisher’s exact test when appropriate. Associations between variables were examined using Pearson correlation test, with Bonferroni correction for multiple comparisons.

## Results

### Characteristics of the patients

This study collected demographic, biochemical, and microbiome data from a total of 320 participants. The cohort comprised 106 healthy controls and 214 patients with MASLD, who were further stratified into DH (n=110) and CD (n=104) pattern groups, as detailed in the participant flowchart (**Figure 1**). Following screening, 214 tongue imaging and coating samples were collected.

Analysis of the demographic characteristics showed that BMI and WHR were significantly higher in MASLD patients than in the control group, indicating increased overall and central obesity. Biochemical analysis demonstrated that glucose metabolism–related indicators, including FPG, 2hPG, HbA1c, FINS, and HOMA-IR, were significantly elevated in MASLD patients compared with controls. In addition, TC, HDL-C levels were significantly altered. Liver function indices, including TBil, DBil, ALT, AST, and ALP, were also significantly increased in MASLD patients.

In the two pattern types of DH and CD patterns, DH pattern showed significantly higher BMI, FINS, and HOMA-IR levels than those with CD pattern. In contrast, CD patients exhibited significantly higher levels of TBil, DBil, ALT, AST, and ALP. No significant differences were observed in the remaining indicators between the two groups. Details are shown in **Table 2**.

**Table 2:** Clinical indicators of participants.

Characteristic	HC(n=106)	DH(n=110)	CD(n=104)	*P*_(HC vs DH vs CD)_	*P*_(DH vs CD)_
Age (years)	60.42 ± 6.25	60.13 ± 5.60	59.12 ± 6.02	0.253	0.205
Gender(male%)	57.50%	60.00%	51.90%	0.477	0.293
BMI(kg/m^2^)	24.19 ± 2.45	27.49 ± 3.85	26.23 ± 3.11	<0.001^***^	0.009^##^
WHR	0.89 ± 0.07	0.98 ± 0.04	0.97 ± 0.06	<0.001^***^	0.107
SBP(mmHg)	125.97 ± 11.03	126.66 ± 11.07	125.51 ± 11.90	0.755	0.464
DBP(mmHg)	78.94 ± 6.95	78.56 ± 8.54	77.34 ± 7.36	0.284	0.261
2hPG(mmo/L)	7.32 ± 1.60	8.60 ± 1.40	8.59 ± 1.40	<0.001^***^	0.946
FPG(mmo/L)	5.51 ± 0.85	5.93 ± 0.66	5.82 ± 0.68	<0.001^***^	0.247
FINS(μIU/mL)	8.06 (6.42-9.65)	15.41 (13.42-17.82)	13.30 (11.47-15.96)	<0.001^***^	<0.001^###^
HOMA_IR	1.96 (1.56-2.36)	4.08 (3.45-4.79)	3.43 (2.88-4.10)	<0.001^***^	<0.001^###^
HbA1c(%)	5.57 ± 0.52	6.00 ± 0.50	6.08 ± 0.51	<0.001^***^	0.284
TC(mmol/L)	4.72 ± 0.57	4.96 ± 0.69	4.87 ± 0.60	0.02^*^	0.295
TG(mmol/L)	1.66 (1.24-2.06)	1.67 (1.34-2.04)	1.47 (1.13-2.01)	0.18	0.089
HDL-C(mmol/L)	1.30 ± 0.22	1.23 ± 0.20	1.24 ± 0.20	0.036^*^	0.546
LDL-C(mmol/L)	2.88 ± 0.53	2.93 ± 0.53	2.82 ± 0.60	0.366	0.168
TBil(μmol/L)	16.42 ± 3.56	17.99 ± 4.90	20.55 ± 5.34	<0.001^***^	<0.001^###^
DBil(μmol/L)	4.49 ± 1.61	5.13 ± 1.59	5.85 ± 1.61	<0.001^***^	0.001
IBil(μmol/L)	13.81 ± 2.11	12.77 ± 5.54	13.71 ± 5.04	0.3	0.193
ALT(U/L)	32.17 ± 21.89	43.84 ± 13.23	51.63 ± 12.51	<0.001^***^	<0.001
AST(U/L)	22.47 ± 7.19	31.97 ± 8.44	35.53 ± 7.95	<0.001	0.002
ALP(U/L)	83.71 ± 19.14	90.18 ± 15.26	96.25 ± 17.62	<0.001	0.008
GGT(U/L)	47.04 (34.67-57.59)	45.75 (33.08-53.70)	44.39 (33.20-52.65)	0.268	0.401
TP(g/L)	72.14 ± 3.65	71.79 ± 3.93	71.71 ± 4.13	0.699	0.889
Cr(μmol/L)	74.12 ± 10.11	75.08 ± 11.76	76.32 ± 12.50	0.381	0.457
UA(μmol/L)	370.45 ± 57.50	371.28 ± 61.87	363.05 ± 64.47	0.563	0.342
eGFR (mL/min/1.73m²)	108.48 ± 7.65	107.80 ± 8.20	107.80 ± 8.20	0.772	0.999

Significant alcohol intake was defined as a daily consumption above 20 g in women and 30 g in men.

*Compared with Control group, *P* < 0.05; **Compared with Control group, *P* < 0.01; #Compared with DH group, *P* < 0.05.

2hPBG, 2-hour postprandial blood glucose; ALP, alkaline phosphatase; ALT, alanine aminotransferase; AST, aspartate aminotransferase; BMI, body mass index; DBiL, direct bilirubin; DBP, diastolic blood pressure; FBG, fasting blood glucose; HbA1c, hemoglobin A1c; HC, hip circumference; HDL-C, high-density lipoprotein cholesterol; IBiL, indirect bilirubin; LDL-C, low-density lipoprotein cholesterol; SBP, systolic blood pressure; TBiL, total bilirubin; TC, total cholesterol; TG, triglycerides; WC, waist circumference; WHR, waist-to-hip ratio; γ-GT, gamma-glutamyl transferase.

### Changes in tongue image features of DH pattern and CD pattern

**Figure 4:**
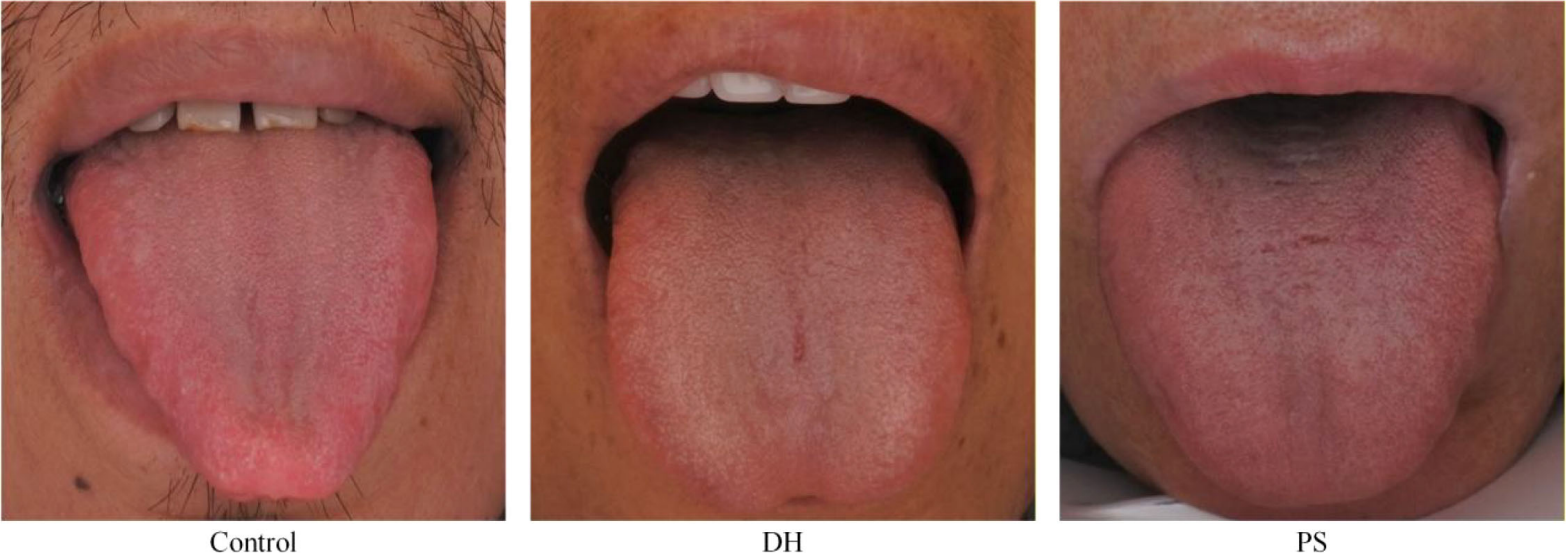
Specific tongue image features of DH group and CD group.

Tongue images were categorized according to expert-defined features, including tongue color (light red, pale, red-crimson), coating color (white, yellow), and morphological characteristics (cracked, teeth-marked, spotted, petechial, greasy, peeling). A chi-square test was performed to compare tongue features between the DH and CD groups (**Table 3**). The results showed that coating color differed significantly between the two groups, with yellow coating being more frequent in the DH group (39.09%), whereas white coating was more common in the CD group (25.00%). In addition, petechial tongue was observed significantly more frequently in the CD group (15.38%) than in the DH group (6.36%). No significant differences were found in tongue color (light red, pale, red-crimson) or other morphological features, including cracked, teeth-marked, spotted, greasy, and peeling fur, between the two groups (**Table 3**). Representative image features are shown in **Figure 4**.

**Table 3:** Tongue features of participants [n (%)].

Feature, n (%).		DH(n=110)	CD(n=104)	χ2 value	*P* value
Tongue color	Light red	55 (50.00%)	58 (55.77%)	0.714	0.398
	Pale	7 (6.36%)	9 (8.65%)	0.405	0.524
	Red-crimson	48 (43.64%)	37 (35.58%)	1.513	0.219
Moss color	White	67 (60.91%)	78 (75.00%)	5.556	0.018^*^
	Yellow	43 (39.09%)	26 (25.00%)	5.556	0.018^*^
Morphological & textural	Cracked	11 (10.00%)	14 (13.46%)	1.503	0.22
	Teeth-print	22 (20.00%)	20 (19.23%)	0.032	0.858
	Spotted	7 (6.36%)	5 (4.81%)	1.154	0.283
	Petechial	7 (6.36%)	16 (15.38%)	11.81	0.001^**^
	Greasy fur	44 (40.00%)	38 (36.54%)	0.287	0.592
	Peeling fur	8 (7.27%)	6 (5.77%)	0.808	0.369

*Compared with DH group, P< 0.05.

**Compared with DH group, P< 0.01.

**Figure 5:**
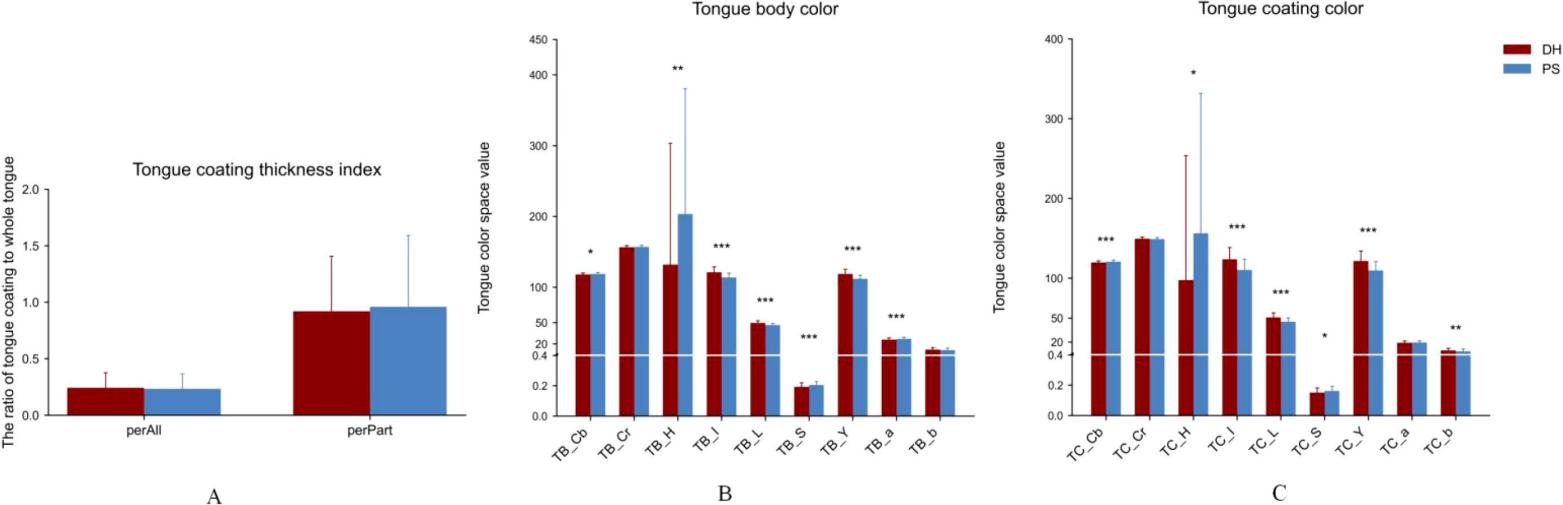
Comparison of tongue image features between the DH and PS groups. **(A)** Tongue coating thickness index. **(B)** Tongue body color features. **(C)** Tongue coating color features. TB: Tongue body; TC: Tongue coating. Tongue coating index: perAll, perPart; The color index is derived from Lab, YCrCb, and HSI color spaces, including L (lightness), a (red–green axis), b (yellow–blue axis), Y (luminance), Cr (red–luminance difference), Cb (blue–luminance difference), H (hue), S (saturation), and I (intensity). *Compared with DH group, P < 0.05; **Compared with DH group, P < 0.01; ***Compared with DH group, P < 0.001.

After extracting quantitative features from tongue images, computer-derived parameters were obtained for both groups. Among the tongue coating thickness indicators, no significant differences were observed between the DH and CD groups in either the overall coating ratio (perAll) or the partial coating ratio (perPart). For tongue body color parameters, significant differences were observed in multiple indices. Compared with the DH group, the CD group showed significantly higher values in TB_H, whereas the DH group exhibited higher values in TB_Cb, TB_Cr, TB_I, TB_L, TB_Y, and TB_a. No significant differences were found in TB_S and TB_b between the two groups. For tongue coating color parameters, several indices also differed significantly between groups. The DH group showed significantly higher values in TC_Cb, TC_Cr, TC_I, TC_L, TC_Y, and TC_a, whereas the CD group had higher values in TC_H and TC_S. No significant difference was observed in TC_a between the two groups. These results indicate that significant differences exist in both tongue body color and coating color parameters between DH and CD patterns (**Figure 5**).

### Oral microbial profiles differed between patients with DH and CD patterns

**Figure 6:**
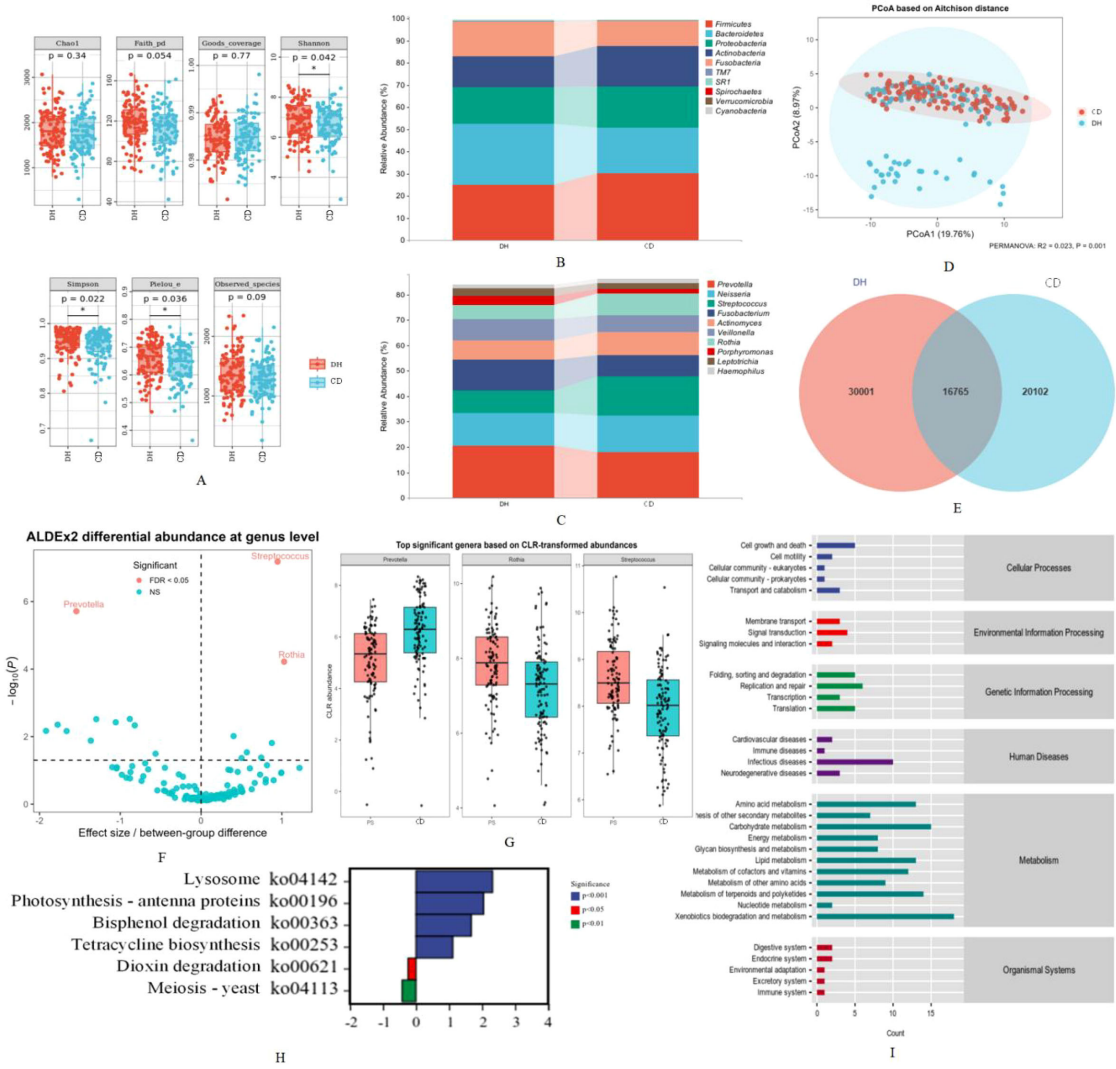
Comprehensive analysis of tongue coating microbiota composition, diversity, and functional profiles between the damp-heat (DH) and cold-dampness (CD) pattern groups. **(A)** Alpha diversity indices of tongue coating microbiota; **(B)** Relative abundance of tongue coating microbiota at the phylum level; **(C)** Relative abundance of tongue coating microbiota at the genus level; **(D)** Principal coordinate analysis (PCoA) based on Aitchison distance showing differences in microbial community structure between groups; **(E)** Venn diagram illustrating shared and unique ASVs between the two groups; **(F)** Differentially abundant genera identified by ALDEx2 analysis; **(G)** Boxplots of key genera based on CLR-transformed abundances; **(H)** Differentially predicted functional pathways between groups; **(I)** Functional annotation of predicted metabolic pathways based on KEGG classification.

According to the results of α-diversity analysis, the Shannon index, Simpson index, and Pielou’s index showed significant differences between the two groups (*P* values were all <0.05), indicating that the tongue coating microbiota in the DH group exhibited higher species diversity and evenness. In contrast, no significant differences were observed in the Chao1 index, Faith’s phylogenetic diversity, or observed species richness between the two groups (**Figure 6A**). Beta diversity analysis based on Aitchison distance and visualized by principal coordinate analysis (PCoA) revealed a partial separation between the DH and PS groups (**Figure 6D**). Permutational multivariate analysis of variance (PERMANOVA) further confirmed that the overall microbial community structure differed significantly between the two groups (R² = 0.023, P = 0.001), although the effect size was relatively small. These results suggest that while the two groups share similar microbial compositions, there are differences in their community structures.

The stacked bar charts showed that, at the phylum level, Firmicutes was the most abundant phylum in both the DH and PS groups (**Figure 6B**). Compared with the DH group, the relative abundance of *Bacteroidetes* exhibited a decreasing trend in the PS group, whereas *Proteobacteria* showed a slight increase (**Figure 6B**). At the genus level, *Prevotella* and *Neisseria* were among the most dominant taxa in both groups (**Figure 6C**). Notably, *Prevotella* showed a relatively higher abundance in the DH group, whereas *Neisseria* tended to be enriched in the PS group. Differential abundance analysis using ALDEx2 revealed significant differences in several genera between the two groups (**Figure 6F**). Genera such as *Streptococcus* and *Rothia* were significantly enriched in the PS group, whereas *Prevotella* was more abundant in the DH group. These differences were further confirmed by boxplot visualization based on CLR-transformed abundances (**Figure 6G**). The Venn diagram demonstrated that the DH and PS groups shared a large proportion of microbial taxa, with 16,765 common features, indicating a substantial overlap in core microbiota between the two groups (**Figure 6E**). Overall, although the two groups shared similar dominant taxa at higher taxonomic levels, compositional differences at the genus level were observed, suggesting distinct microbial signatures associated with different TCM patterns.

The preceding analyses mainly addressed the diversity and taxonomic composition of the microbial community. However, in microbial ecology, characterization of the functional potential of microbial communities is equally essential. Therefore, we further conducted statistical analyses of metabolic pathways. The results showed that microbial functions were mainly enriched in metabolic pathways, including amino acid metabolism, carbohydrate metabolism, lipid metabolism, and xenobiotics biodegradation and metabolism in both DH and CD patterns (**Figure 6I**). In addition, compared with the DH group, the CD group showed down-regulation of pathways related to cellular processes and energy metabolism (Lysosome, Photosynthesis - antenna proteins, Bisphenol degradation, Tetracycline biosynthesis), and up-regulation of pathways related to detoxification and the cell cycle (Dioxin degradation, Meiosis - yeast) (**Figure 6H**).

### Correlation of oral microbiota with tongue features

**Figure 7:**
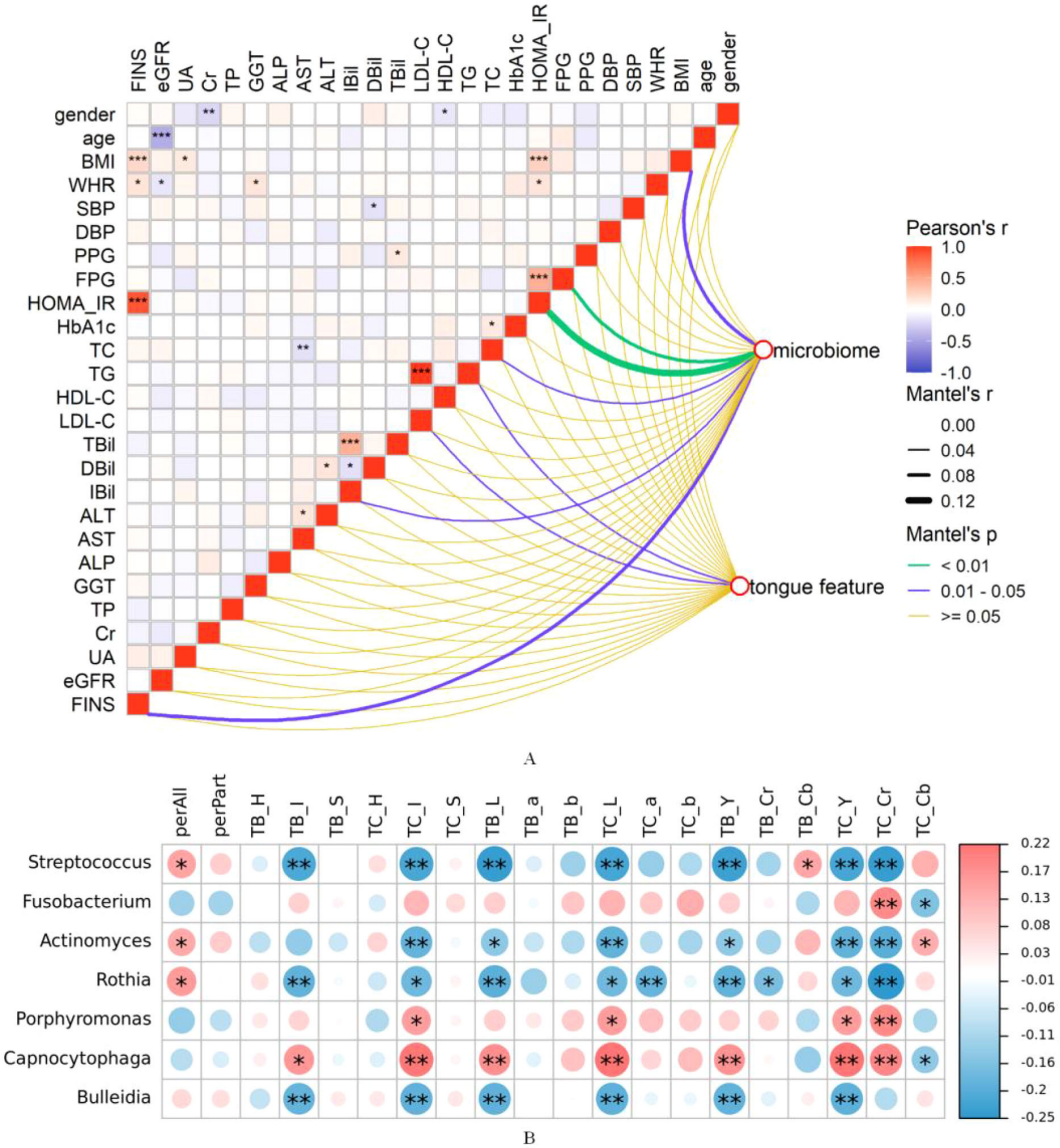
Correlation analysis among tongue coating microbiota, tongue image features, and clinical indicators. **(A)** Mantel test assessing the associations among tongue image features, tongue coating microbiota, and clinical indicators; **(B)** Heatmap showing Pearson correlation coefficients between tongue coating microbiota and tongue image features.

Through mental-test analysis, we confirmed that the characteristics of the tongue image were correlated with the body mass index (BMI) and the liver function index ALT (**Figure 7A**). Meanwhile, the tongue coating microbiota is associated with body mass index (BMI) and indicators related to glucose and lipid metabolism and insulin resistance (FPG, HBA1c, HOMA-IR, FINS) (**Figure 7A**). Additionally, through Pearson correlation analysis, we confirmed that the tongue coating microbiota is correlated with the imaging changes of the tongue. Among them, *Streptococcus* and *Rothia* in the tongue coating microbiota were positively correlated with perAll, and negatively correlated with TB_I, TC_I, TB_L, TC_L, TB_Y, TC_Y, and TC_Cr, which represent the color and brightness of the tongue body and tongue coating. Conversely, *Capnocytophaga* was positively correlated with TB_I, TC_I, TB_L, TC_L, TB_Y, TC_Y, and TC_Cr, which represent the color and brightness of the tongue body and tongue coating. The heatmap shows the Pearson correlation coefficients between the tongue image indicators and the top 10 species at the genus level in the relative abundance of the oral microbiome, as shown in **Figure 7B**.

**Figure 8:**
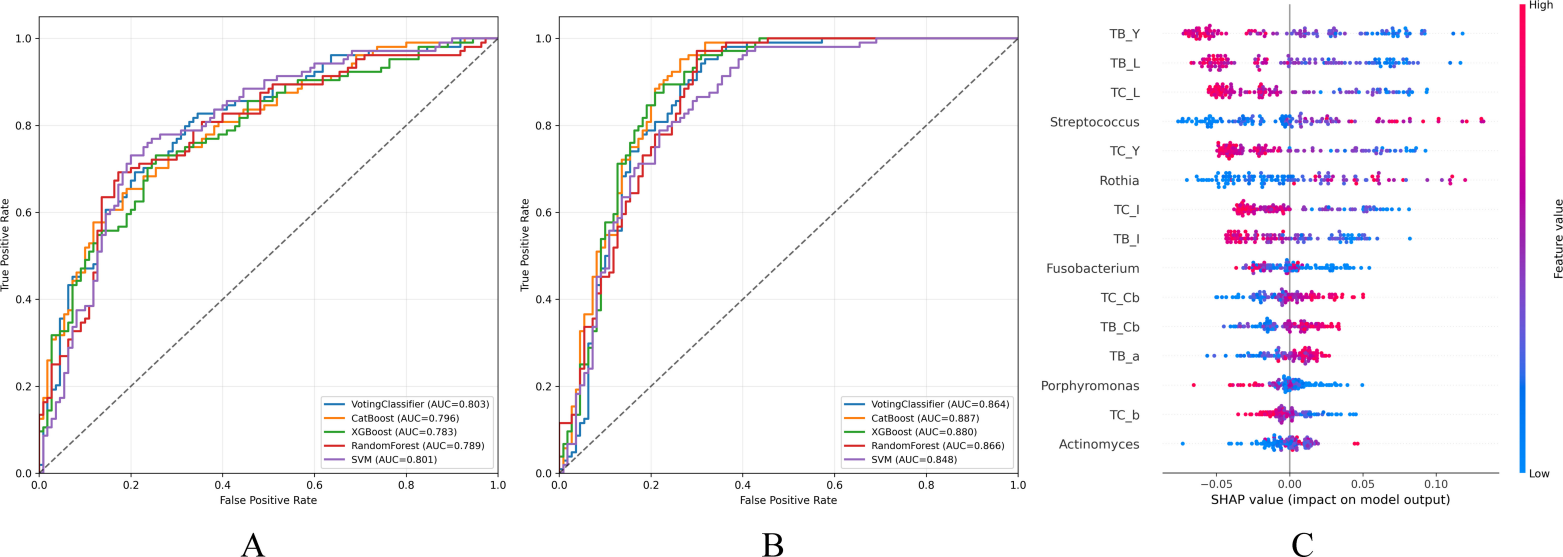
Construction of classification models for distinguishing damp-heat (DH) and cold-dampness (CD) pattern groups. **(A)** Receiver operating characteristic (ROC) curve of the classification model based on tongue image features; **(B)** ROC curve of the classification model integrating tongue image features and tongue coating microbiota; **(C)** SHAP summary plot illustrating feature contributions to the model.

Construction of a pattern classification model for DH and CD patterns using machine learning Building upon preliminary research, we identified significant differences in tongue image characteristics between the two patterns, along with distinct signature microbiota present in their tongue coatings. Consequently, we aimed to develop a classification model for MASLD patterns centered on tongue image characteristics. The combination of tongue image features and signature tongue coating microorganisms demonstrated superior efficacy in classifying the two patterns compared to tongue image characteristics alone. When only tongue image indicators were used for modeling, the best diagnostic performance was achieved by support vector machine (SVM), with an area under the curve (AUC) of 0.802 and an accuracy rate of 0.734 (**Figure 8A**), indicating that the imaging features of the tongue can effectively distinguish DH pattern from CD pattern. After including Streptococcus, Rothia, and Porphyromonas, the performance of all models improved. Among them, the random forest model performed the best, with the accuracy rate increasing to 0.790 and the AUC value reaching 0.871 (**Figure 8B**, **Table 4**). According to results of the random forest feature importance assessment indicated that among the tongue image parameters, TB_Y, TB_L, and TC_L, which represent the color and lightness of the tongue body and tongue coating, had the highest contribution to the prediction results. Next were Streptococcus and Rothia in the tongue coating microbiota (**Figure 8C**).

**Table 4:** Diagnostic performance comparison of five machine learning models.

Variables	Model	AUC	Accuracy	Precision	Recall	F1-score
Tongue	RandomForest	0.7872	0.6963	0.6757	0.7212	0.6977
	SVM	0.8024	0.7336	0.7043	0.7788	0.7397
	XGBoost	0.7508	0.7056	0.6952	0.7019	0.6986
	LogisticRegression	0.7895	0.715	0.6972	0.7308	0.7136
	LightGBM	0.7395	0.7009	0.6961	0.6827	0.6893
Tongue & Tongue coating microbiome	RandomForest	0.8709	0.7897	0.7565	0.8365	0.7945
	SVM	0.8481	0.7804	0.7664	0.7885	0.7773
	XGBoost	0.8624	0.7991	0.785	0.8077	0.7962
	LogisticRegression	0.8113	0.757	0.7364	0.7788	0.757
	LightGBM	0.8594	0.785	0.7788	0.7788	0.7788

## Discussion

Pattern differentiation is the cornerstone of TCM diagnosis and treatment (7). Dampness patterns are predominant in MASLD. Distinguishing whether patients with dampness patterns present with damp-heat or cold-dampness is a clinically crucial issue. However, the relative predominance of cold and heat within dampness patterns is difficult to determine by visual inspection alone. Based on this reason, this study objectively and quantitatively described the tongue image of MASLD patients with DH pattern and CD pattern. It was found that there were differences in objective indicators between the tongue image of MASLD patients with DH pattern and CD pattern, and at the same time, it was found that the tongue coating microorganisms were different between DH pattern and CD pattern. The combination of tongue image objective indicators and tongue coating microorganisms can effectively classify DH pattern and CD pattern in MASLD patients.

TCM pattern patterns often evolve with disease progression (26). Clinical parameters indicated that patients in the DH group exhibited higher BMI and more pronounced insulin resistance, whereas those in the CD group demonstrated more obvious hepatic dysfunction and cholestasis. Research has shown that the progression of MASLD disease gradually shifts from disorders of glycolipid metabolism to liver function impairment (27, 28). In light of the findings of this study, we tentatively believe that patients with damp-heat and cold-damp patterns exhibit distinct characteristics in metabolic and liver function indicators; the patterns of their dynamic evolution require verification through subsequent longitudinal cohort studies. Although differences exist between the two patterns, these features are relatively subtle and often clinically occult, requiring invasive examinations for accurate identification in clinical practice.

Tongue diagnosis, regarded as the “window of internal organs,” provides important information for pattern differentiation and treatment in traditional Chinese medicine (29). In the present study, although inspection diagnosis failed to identify significant differences DH pattern and CD pattern, objective tongue image analysis based on quantitative indicators was able to effectively distinguish between these two patterns, especially TB_I, TB_L, TB_Y, TB_Cr, TC_I, TC_L, TC_Y, TC_Cr. Specifically, patients with DH pattern tended to present with a relatively red tongue and a yellow greasy coating, which is similar to findings reported in patients with ulcerative colitis with Damp-Heat pattern (30). In contrast, patients with CD pattern typically exhibited a dull tongue color, a white greasy coating, and the presence of ecchymotic spots. From the perspective of TCM theory, the DH pattern is mainly associated with the predominance of internal heat, whereas the CD pattern reflects the coexistence of dampness, cold, often accompanied by yang deficiency (12). Meanwhile, the changes in tongue image also have certain biological implications. Previous studies have shown that abnormal expression of lipids and lipid-like molecules in greasy tongue coatings is closely associated with disturbances in glycerophospholipid metabolism (31). In addition, tongue color may, to some extent, reflect vascular elasticity and blood oxygen saturation (32).

Changes in tongue color and coating are closely associated with the tongue coating microbiome (15, 33), alterations in the microbiota composition of the tongue coating may influence tongue image (49), both of them are related to TCM patterns (9). Our study found that tongue coating microbiota differed in composition and abundance between the two MASLD patterns. This demonstrates that different TCM pattern types within a single disease harbor distinct microbiological features, thereby validating the principle of pattern classification from a microecological standpoint. In terms of microbial composition, *Prevotella* and *Neisseria* are the dominant genera in DH and CD, with *Prevotella* being more enriched in DH and *Neisseria* being more enriched in CD. *Prevotella*, a Gram-negative anaerobe, is known to promote inflammatory responses through lipopolysaccharide (LCD) production and immune modulation (34, 35). *Neisseria* is more often linked to a relatively homeostatic oral microbial pattern (36). *Prevotella*, *Streptococcus* and *Rothia* may be the key strains to distinguish the DH pattern type from the CD pattern type, which is likewise manifested in different patterns among patients with disease of digestive tract (37, 38). *Streptococcus* has been reported to be associated with MASLD severity and liver injury indicators, including ALT and AST levels, and may influence hepatic lipid metabolism and hepatocellular damage (39, 40). The differences in the enrichment trends of oral microbiota between DH and CD also align with the clinical indicators of these two pattern types. Specifically, the DH pattern presents a pathological state mainly characterized by inflammation and metabolic disorders, while the CD pattern is marked by abnormal liver function.

Our results showed that the tongue coating microbiota was closely associated with glucose and lipid metabolism as well as insulin resistance-related indices, whereas tongue image features were more strongly correlated with BMI and liver function indicators. This finding is consistent with previous studies demonstrating that oral microbiota is linked to host metabolic status and participates in metabolic disorders through the oral–liver axis (41, 42). In contrast, tongue features, as external manifestations of internal physiological conditions, have been reported to reflect metabolic status and liver function alterations (43). Moreover, we observed a close association between objective tongue image parameters and the tongue coating microbiota. This may be related to the colonization of the oral microbiota and the adhesion and biofilm formation of microorganisms (44), and affects the stability of the oral micro-environment (45). Meanwhile, oral microbiota can induce local inflammatory responses in the oral cavity and subsequently affect local vascular responses and pigment-related changes, thereby leading to alterations in the redness, yellowness, and brightness of the tongue. These correlations all suggest that changes in tongue image may be closely related to alterations in microbial composition and local microecological status. Pathway analysis revealed that the majority of tongue coating microbiota associated with DH and CD patterns were enriched in metabolism-related pathways, which is consistent with previous studies showing that oral and gut microbiota in metabolic disorders such as MASLD are predominantly involved in lipid metabolism and energy metabolism pathways (46). Furthermore, differential functional pathway analysis indicated significant differences between the CD and DH groups. Compared with the CD group, the DH group exhibited upregulation of pathways related to cellular processes and energy metabolism, which is consistent with the research results that the microbiota shows enhanced energy production under inflammatory conditions (47). Whereas the CD group showed upregulation of pathways associated with detoxification and the cell cycle.

This study established a pattern classification method based on objective tongue diagnostic features, achieving good predictive performance with an AUC of 0.803, and further supporting the important role of tongue diagnosis in pattern differentiation. Moreover, integrating objective tongue features with tongue coating microbiome data through a machine learning approach markedly improved classification performance, enabling effective discrimination between DH and CD patterns in MASLD patients, with an AUC of 0.887. The research results indicate that the combination of tongue images and microbiota can effectively distinguish different pattern types of diseases, which is consistent with the research results of Jiang et al. on the differentiation of pattern types of chronic gastritis (48). Among the tongue features, TB_L and TC_L, which reflect the brightness of the tongue body and coating, as well as YB_Y and YC_Y, which represent the yellow chromatic components of the tongue body and coating, played pivotal roles in classification. In addition, our findings further support the potential of oral microbiota as biomarkers for disease and pattern diagnosis as reported in previous studies (49, 50). Specifically, *Streptococcus* and *Rothia* showed significant contributions to the classification model. Compared with traditional laboratory indicators, tongue coating microbial markers demonstrate unique advantages. Compared with traditional laboratory indicators, tongue coating microbial markers exhibit unique advantages. Tongue image interpretation is prone to human subjectivity, circadian rhythm (51), and environmental factors, posing challenges to their stability and objectivity (52). In contrast, tongue coating microbiota can be collected noninvasively and measured objectively, providing relatively stable biological information under standardized sampling conditions (53). Therefore, converting key microbial markers into clinically usable objective tools is a key direction for future research.

However, this study has several limitations. First, the sample size was relatively small, and larger cohorts are needed to better control for potential confounding factors. Second, as the study population was derived from specific geographic regions and demographic backgrounds, the generalizability of the findings may be limited, warranting validation in more diverse populations. Third, this study focused on two primary patterns, and future studies should include additional patterns, such as blood stasis and liver depression with spleen deficiency. In future work, we plan to integrate gut microbiota to develop diagnostic models based on microbial signatures. Additionally, metabolomics analyses will be conducted to elucidate the biological mechanisms underlying tongue features, thereby further improving the objectivity and clinical applicability of tongue-based MASLD pattern classification.

In conclusion, this study revealed differences in disease severity, tongue image features, and tongue coating microbiota between the “Damp Heat” and “Cold dampness” patterns and further explored the associations between tongue image characteristics and microbial communities, and evaluated the potential of tongue coating microbiota as auxiliary biomarkers for pattern classification. Our findings provide preliminary evidence linking tongue image characteristics with microbial metabolism, offering a theoretical basis for the application of tongue diagnosis in TCM pattern differentiation.

## Data Availability

The datasets presented in this study can be found in online repositories. The names of the repository/repositories and accession number(s) can be found in the article/supplementary material.
